# Preclinical approaches in chronic myeloid leukemia: from cells to systems

**DOI:** 10.1016/j.exphem.2016.11.005

**Published:** 2017-03

**Authors:** Cassie J. Clarke, Tessa L. Holyoake

**Affiliations:** Paul O'Gorman Leukaemia Research Centre, Institute of Cancer Sciences, University of Glasgow, Gartnavel General Hospital, Glasgow, UK

## Abstract

Advances in the design of targeted therapies for the treatment of chronic myeloid leukemia (CML) have transformed the prognosis for patients diagnosed with this disease. However, leukemic stem cell persistence, drug intolerance, drug resistance, and advanced-phase disease represent unmet clinical needs demanding the attention of CML investigators worldwide. The availability of appropriate preclinical models is essential to efficiently translate findings from the bench to the clinic. Here we review the current approaches taken to preclinical work in the CML field, including examples of commonly used in vivo models and recent successes from systems biology-based methodologies.

## Highlights

• CML is a hematopoietic stem cell disorder caused by a chromosomal translocation.
• TKIs have transformed CML treatment but do not eradicate LSC which maintain disease.
• The current preclinical approaches applied in CML research are reviewed.
• Successfully combining approaches to deliver preclinical packages is discussed.

**Figure 1:**
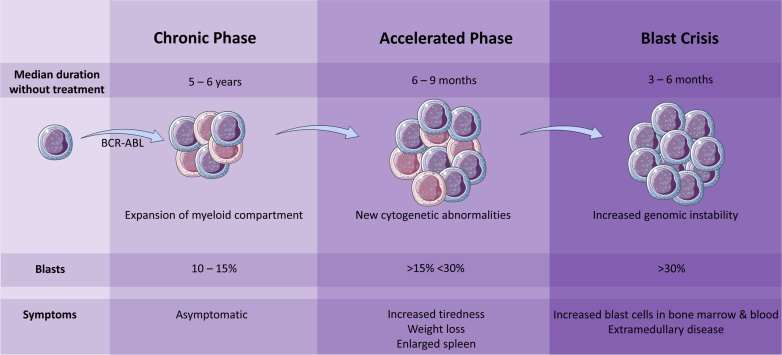
CML disease progression. The majority of CML patients are diagnosed in the chronic phase, which will progress through the accelerated phase to blast crisis if untreated. Each phase can be characterized by the number of immature cells (blasts) found in the BM. Expression of BCR-ABL activates a number of signaling pathways, resulting in increased proliferation and decreased apoptosis in the myeloid compartment. Secondary genetic and molecular abnormalities lead to an accumulation of mutations and genomic instability, resulting in progression to blast crisis and poor patient prognosis.

Chronic myeloid leukemia (CML) is a myeloproliferative disorder of hematopoietic stem cell (HSC) origin caused by the t(9;22) chromosomal translocation. Fusion of part of the breakpoint cluster region (BCR) on chromosome 22 with the Abelson murine leukemia viral oncogene homolog 1 (ABL) tyrosine kinase of chromosome 9 results in formation of the Philadelphia (Ph) chromosome and expression of the BCR-ABL fusion gene. The consequent BCR-ABL protein generated is a constitutively active tyrosine kinase that can influence a number of major signaling pathways involved in cell survival, proliferation, adhesion, and differentiation. Clinically this presents as an expansion of myeloid cells and an accumulation of differentiating granulocytic precursors and differentiated effector cells, with increased peripheral granulocytosis, splenomegaly, thrombocytosis, and anemia. The majority of patients tend to present with chronic phase disease (CP), which, without therapeutic intervention, then proceeds through to an accelerated phase (AP) and ultimately to blast crisis (BC) over approximately 3 to 5 years (Fig. 1). The initial dependence of CML on the expression of BCR-ABL has led to the development of a number of ABL tyrosine kinase inhibitors (TKIs), with such TKIs now representing the front-line therapy for CML patients.

Imatinib, approved by the Food and Drug Administration (FDA) in 2001, was the first TKI brought to the market and approved for use in CML patients. By attaching closely to the ATP binding site of BCR-ABL, this first-generation TKI stabilizes the inactive conformation of BCR-ABL, thus inhibiting its constitutive tyrosine kinase activity. Subsequent second-generation TKIs have since been developed to combat the problem of imatinib resistance, including dasatinib, bosutinib, and nilotinib, and all are now approved as first-line treatments for newly diagnosed CML patients, with the exception of bosutinib, which tends to be prescribed only when imatinib, dasatinib, and nilotinib are not viable options. However, whether because of side effects, noncompliance, or TKI resistance, TKIs fail a significant proportion of patients, meaning that the development of new therapies for treatment of CML is still a priority. There are therefore still a number of areas of unmet clinical need in the CML field that require attention, and so to efficiently translate findings from the laboratory to patients, it is essential to have effective preclinical models in place that are predictive of performance in the clinic.

## In vitro models

### Immortalized hematopoietic cell lines

Hematopoietic cell lines expressing the Ph chromosome provide a basic system in which to assess the molecular effects and responses of a CML-like cell (Table 1). Offering a continuous source of reproducible cellular material and being amenable to an exhaustive number of in vitro assays, immortalized cell lines are frequently used as an initial high-throughput tool to validate potential therapeutic targets and screen drug candidates. High-throughput screens (HTS) have been instrumental in the development of CML therapies, with the work that led to the discovery of imatinib stemming from HTS-identified kinase inhibitors [1]. The ease with which cell lines can be manipulated also enables their utilization in a variety of ways, including genomewide RNA interference screens to identify TKI-resistant genes [2], and reporter gene assays to provide further information regarding the specificity of signaling pathways involved [3]. Furthermore, techniques that involve use of multiple constructs, such as optimization of short interfering RNA/small hairpin RNA-based approaches, would not be possible in limited amounts of primary patient material, and so use of immortalized cell lines in such instances is also essential. However, immortalized cell lines are often derived from patients in BC and, so, frequently contain further mutations in addition to expression of the BCR-ABL fusion product. Excessive culture in the hands of different research groups can also mean that the same cell line in different laboratories may ultimately become genetically distinct, and results generated in such systems are not necessarily indicative of in vivo responses. Nevertheless, immortalized hematopoietic cell lines are frequently used in early-stage projects to form the basis of subsequent preclinical work, and gene expression analysis of 40 hematologic cell lines revealed that the vast majority of cell lines do indeed cluster according to clinical and molecular subtype based on their gene expression profile [4], indicating that they do, to a certain extent, retain the appropriate pathways of interest. An initial review of CML-derived cell lines indicated early support for the hypothesis that the Ph translocation is not restricted to lineage-committed progenitor cells [5], and ABL-specific TKIs are consistently effective against human CML and Ph^+^ cell lines in vitro because of their dependence on BCR-ABL for proliferation and survival 6, 7. Subsequent studies in BCR-ABL-expressing cells also predicted that continuous suppression of BCR-ABL would be necessary for clinical benefit in CML [8], and therefore, although data generated in immortalized hematopoietic cell lines should always be treated with caution, their utility should not be underestimated.

### Primary patient CML cells

The use of primary patient samples, comparing CML cells with normal hematopoietic progenitors, is an essential component of the preclinical CML package, combining the relevance of variable patient biology with in vitro assays to understand the molecular basis of disease and to indicate responses to potential therapies. Because primary material cannot be manipulated to the same extent as immortalized cells, the assays amenable for use in such samples are somewhat more limited than those applied in immortalized cell lines. However, there are still a large number of approaches available for use in primary samples that can provide a wealth of information, including colony-forming cell and long-term culture-initiating cell assays, in addition to flow cytometry, cell cycle and apoptosis, Western blot, immunofluorescence, and polymerase chain reaction approaches (Table 2). Improvements across “omics” approaches mean that these powerful techniques are also now possible on smaller amounts of starting material, even down to the single-cell level in some instances, with such platforms opening up a number of possibilities in understanding deregulated networks in disease and drug treatment 9, 10.

Biobanks of such patient samples are an invaluable source to researchers, and cells are often processed, banked, and used as defined cell populations, such as CD34^+^ and CD34^+^CD38^−^ sorted cells, with CD34^+^ enrichment being used as a bare minimum to deplete the mature granulated cells that are numerous in CML and, when present, cause technical issues on degranulation during freeze–thaw. Use of dual-fluorescence in situ hybridization (D-FISH) for BCR-ABL is also necessary to distinguish normal versus CML cells that coexist within the same stem cell compartment of patients. Once CML CD34^+^ cells of interest are isolated and placed in liquid culture with appropriate supporting growth factors, difficulties encountered in vitro include expanding the primitive cells of interest and their short window of use, with CP cells having a tendency to mature when cultured [11]. Despite these technical limitations, the use of primary CML material in vitro has been instrumental in developing our understanding of CML cells and their response to therapy. Although TKI resistance is a well-recognized limitation in the treatment of CML 12, 13, leukemic stem cell (LSC) persistence also underlies our current inability to cure the disease. While, in the absence of drug resistance, TKIs are effective against the majority of CD34^+^CD38^+^ and CD34^+^CD38^−^ cells in CML patients 14, 15, more primitive LSCs are much less susceptible to the apoptosis induced by TKI treatment, with their persistence maintaining disease 16, 17, 18, 19. Data derived from human primary samples therefore indicate that LSC survival is not dependent on BCR-ABL kinase activity 20, 21, and curative therapies in CML will thus have to target additional pathways.

The comparison of primary cell populations derived from normal donors versus CML patients has implicated a large number of molecular pathways in LSC survival, with a number of these pathways being reviewed concisely elsewhere 22, 23. Combined approaches targeting BCR-ABL and additional targets in such pathways have thus been investigated in CML CD34^+^ cells. These include studies assessing the effects of the Janus kinase 2 inhibitors, which indicated that reduced activity of the Janus kinase 2/signal transducer and activator of transcription 5 pathway, in combination with BCR-ABL inhibition by nilotinib, was able to increase apoptosis of CML stem/progenitor cells, both in vitro and in vivo 24, 25, 26, 27. Furthermore, activation of peroxisome proliferator activated receptor gamma by the anti-diabetic drug pioglitazone was also able to work synergistically with TKI to deplete CML LSCs through decreasing expression of signal transducer and activator of transcription 5 and its downstream targets [28]. Altered transforming growth factor beta-forkhead box signaling has also been found in LSCs following TKI exposure [29], with transforming growth factor beta inhibitors being effective in reducing colony formation in vitro [30], while a number of studies have implicated the Hedgehog pathway in LSC persistence 31, 32, 33 with consequent use of a smoothened (SMO) inhibitor, in combination with TKI, reducing CD34^+^ CP-CML cell engraftment in vivo [33]. At the epigenetic level, use of histone deacetylase inhibitors, in combination with TKI, have been shown to successfully target quiescent LSCs [34], while enhancer of zeste homolog 2 (EZH2) and H3K27me3 reprogramming have also been found to be important for LSC survival, with consequent use of EZH2 inhibitors in combination with TKI also providing a promising therapeutic means of eradicating LSCs [10]. The contribution of cellular processes, in addition to specific signaling pathways, to LSC survival has also been probed, including targeting autophagy and lipid metabolism to induce cell death in Ph^+^ primary CML stem cells 35, 36, 37. And so, use of primary CML patient cells in vitro has been essential in understanding which dual targeting approaches may be successful going forward to the clinic.

## In vivo models

Although the use of in vitro techniques is a necessary prerequisite to all projects that progress to generate therapeutic opportunities, in vivo models have the major advantage of providing a more relevant microenvironment, recapitulating interactions between different cell types under more appropriate physiologic conditions. There are currently three commonly used mouse models applied in CML research (Table 3), with each having its own advantages and caveats, and each being appropriate in its own right depending on the hypothesis being tested.

### Retroviral transduction/transplantation model

**Figure 2:**
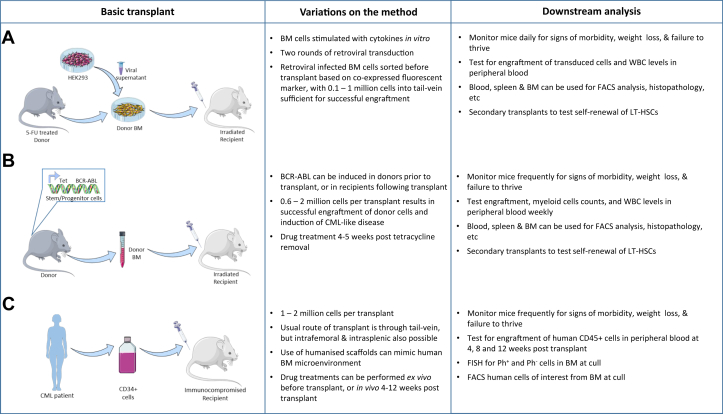
Schematic representation of the three mouse models of CML. The three main mouse models of CML are described, including variations described in the literature and commonly performed downstream analysis. (**A**) Retroviral model: Donor mice are treated with 5-FU; BM cells are collected and treated with BCR-ABL retrovirus produced from HEK293 cells; and transduced cells are transplanted into irradiated recipients. (**B**) SCLtTA/BCR-ABL transgenic model: BM is isolated from SCLtTA/BCR-ABL donors and transplanted into irradiated recipients, with BCR-ABL expression being inducibly expressed in the stem and progenitor cells on removal of tetracycline from the drinking water. (**C**) Xenograft model: CD34^+^ CML cells are isolated from patient material and transplanted into immunocompromised recipient mice.

The murine retroviral model involves infection of 5-fluorouracil (5-FU) treated mouse bone marrow cells with retrovirus encoding BCR-ABL, followed by transplantation of transduced cells into irradiated syngeneic recipients 38, 39, 40 (Fig. 2A). Recipients develop hematologic malignancies, including a myeloproliferative CML-like disease resembling CP human CML. Although these studies established that BCR-ABL in such a system can cause myeloproliferative disease in mice, in these initial models more than one type of disease developed, whereas the leukemic phenotype observed was also dependent on how donor mice were conditioned, suggesting that the target cell in which BCR-ABL was expressed, and additional mutations in those cells, was important in determining the disease burden [41]. Furthermore, using the original published methods, not all recipients developed CML-like disease with a consistent latency. As a consequence, a number of variants of the retroviral model, including use of modified retroviral constructs, different viral packaging systems, and alternative viral infection methods, have since been described in the literature, resulting in a frequently used model with faithful development of CML-like syndrome in 100% of recipients 2–4 weeks post transplantation 52, 53. The rapid onset of disease in this model means that it is much more aggressive than the typical progression of CML in human patients; however, in an experimental setup, this can also have it advantages, allowing efficient feedback to evaluate the therapeutic effects of agents tested in vivo. The retroviral method is also an effective method to monitor the effects of co-expression or deletion of other genes of interest, and their variants, with data generated in this model revealing that expression of interferon regulatory factors, for example, can act in a tumor suppressor role to regulate proliferation of normal and leukemic hematopoietic cells, with consequent overexpression of such factors inhibiting myeloproliferative disorder [54]. Recent work has also indicated that the scaffolding adaptor protein GRB2-associated binding protein 2 is required for BCR-ABL-induced leukemogenesis [55], and that IκB kinase-dependent activation of NF-κB can also contribute to Ph^+^ leukemias [56], whereas others have used the same model to illustrate that targeting methyltransferases, such as protein arginine methyltransferase 5 (PMRT5), can eliminate LSCs [57], thus identifying additional potential therapeutic targets of interest. Furthermore, it is also an ideal system in which to understand the effect of mutant variants of BCR-ABL, with studies indicating that although T315I and Y253H mutations of BCR-ABL confer resistance to imatinib, they do not provide a growth advantage in the absence of TKI [58]. The effects of therapeutics can thus be tested in a setting that compares the effects of wild-type (WT) and mutant BCR-ABL, with treatment with a HSP90 inhibitor indicating that HSP90 could be a therapeutic target for CML induced by either WT or T315I BCR-ABL 52, 59. Triple-gene-expression systems can also be effectively used in the retroviral model, designing constructs to simultaneously express BCR-ABL, Cre recombinase (Cre), and GFP to enable assessment of BCR-ABL in GFP-selected cells for a conditional Cre-induced knockout of interest. In such cases, deletion of the tumor suppressor gene phosphatase and tensin homolog (PTEN) was found to accelerate the development of CML, as recipient mice receiving cells transduced from bone marrow (BM) of Pten^fl/fl^ mice with BCR-ABL-Cre-GFP retrovirus developed CML at a faster rate than those receiving cells transduced with the corresponding BCR-ABL-GFP control retrovirus, while overexpression of PTEN consequently delayed CML development [52]. Gene expression can therefore be manipulated with ease in the retroviral transduction/transplantation model, and so this murine approach is an effective means of assessing the effects of specific targets of interest, their interaction with BCR-ABL, and their responses to targeted therapies.

### SCLtTA/BCR-ABL transgenic model

In the SCLtTA/BCR-ABL transgenic mouse model of leukemogenesis the expression of P210 BCR-ABL is regulated by a tetracycline-controlled transactivator (tTA) under the control of the murine stem cell leukaemia gene 3’ enhancer (SCL), specifically driving expression of BCR-ABL in the stem and progenitor cells of the hematopoietic system when tetracycline is removed from the drinking water [42] (Fig. 2B). Clinical characteristics following BCR-ABL expression in this model include neutrophilia, leukocytosis, and invasion of myeloid cells into multiple organs, including the liver, lungs, and lymph nodes, thus also conferring a CML-like disease to the mice following BCR-ABL expression. Although survival following BCR-ABL expression varies, on average, from 4 to 10 weeks, the model does result in a consistent expansion of the HSC and myeloid compartments of the BM and an increase in the number of progenitor cells in the spleen. As opposed to the fast and dramatic onset of disease in the retroviral model, this system has a natural progression that is more similar to that of human CML and, thus, may provide a more physiologically relevant model for studying the events that initially follow BCR-ABL expression [42]. Furthermore, the phenotype is also transplantable in sublethally irradiated syngeneic recipients, via both Lin^−^Sca-1^+^c-kit^+^ (LSK) cells and unfractionated BM, with transplant of unfractionated BM generating a more severe disease phenotype, presumably because of the presence of supporting cells. Leukemic spleen cells are also capable of transplanting disease, with expression of BCR-ABL in such cells having cell-autonomous effects that consequently affect their engraftment potential and response to TKI [44]. Studies based on this model have reinforced the understanding that the inhibition of BCR-ABL in vivo does not eliminate the LSKs that maintain disease, and have also indicated that BCR-ABL expression induces differentiation and decreases the self-renewal capacity of the LSK population 21, 44. Gene expression profiling in the LSK population from BCR-ABL^+^ and control mice revealed that BCR-ABL expression induced an increase in expression of genes involved in proliferation and myeloid differentiation, whereas genes involved in self-renewal were downregulated [43]. The contribution of genomic instability to disease progression has also been investigated, with elevated levels of reactive oxygen species-induced DNA damage being found in LSCs, which was consequently able to generate a number of clinically relevant mutations in BCR-ABL and additional tumor-promoting factors [60]. The SCLtTA/BCR-ABL model has also been used to explore the role of the microenvironment in CML development, with studies indicating that leukemic myeloid cells can remodel the endosteal BM niche into an environment that impairs normal hematopoiesis and promotes leukemic progression [61], and that specific cytokines produced by leukemic cells can influence the BM microenvironment to provide favorable conditions for CML LSC growth [62]. This transgenic system is thus a suitable model in which to assess the effects of therapies on long-term HSC (LT-HSCs) in an appropriate in vivo microenvironment.

Further derivations of the SCLtTA/BCR-ABL model have also been generated. To recapitulate progression to BC in an unbiased in vivo setting, Giotopoulos et al combined the SCLtTA/BCR-ABL double transgenic model with a transposon-based insertional mutagenesis system to generate a murine model of CML progression [63]. The addition of a transposition element to this model, mimicking the additional chromosomal instability and mutagenesis occurring in BC, resulted in a change in terminal kinetics of the disease and an increase in myeloid leukemogenesis. Analysis of common insertion sites revealed disruption of a number of genes with known associations with CML progression, particularly genes involved in transcriptional regulation of the stem cell compartment, and progression to BC in the model displayed gene expression profiles comparable to those of human BC. The BC-associated signature included increased expression of c-Myc and its targets, suggesting that activation of this pathway is important during BC progression. This was further reinforced by the ability of I-BET (an inhibitor effective against bromodomain and extraterminal [BET] proteins, which are critical in mediating Myc transcription), to decrease clonogenic growth in both the mouse model and patient samples. However, it was also interesting to note that the comparison of insertion sites between CML-BC mice and mice harboring only the transposition element differed, indicating that BCR-ABL may influence mutational selection pressure. Thus, in addition to identifying and testing potential therapeutic targets of interest, evolution of existing disease models can also provide additional information on the molecular progression of disease processes.

### Xenograft model

In the xenotransplant model of CML human malignant cells are engrafted into immunocompromised mice, with the subsequent frequency of LSCs, and their long-term Ph^+^ engraftment ability, then being assessed. Although a number of different mouse strains are amenable for use, including NOD.Cg-Prkdc^scid^/IL2rg^tm1Wjl^/SzJ (NSG) and NOD.Cg-Prkdc^scid^Il2rg^tm1Sug^/JicTac (NOG) mice, the common theme among them is their immunodeficient status, which facilitates engraftment of human cells 64, 65. Additional strains are now becoming commercially available with the purpose of facilitating increased engraftment of human cells, with examples including the NOD.Cg-Prkdc^scid^Il2rg^tm1Wjl^Tg(CMV-IL3,CSF2,KITLG)1Eav/MloySzJ (NSG-SGM3/NSGS) [66] and Rag2^tm1.1Flv^Csf1^tm1(CSF1)Flv^Csf2/Il3^tm1.1(CSF2,IL3)Flv^Thpo^tm1.1(TPO)Flv^Il2rg^tm1.1Flv^Tg(SIRPA)1Flv/J strains (MISTRG, with a MITRG strain also available that does not include the BAC-transgene encoding human SIRPα) [67], all of which transgenically express human cytokines with the aim of improving human cell engraftment, and the NOD.Cg-Kit^W-41J^Tyr^+^Prkdc^scid^Il2rg^tm1Wjl^/ThomJ (NBSGW) strain, where a Kit mutation is proposed to facilitate the engraftment of human hematopoietic cells without the standard requirement of irradiation [68]. As appreciation of the role of the microenvironment in leukemia development grows [50], implantation of scaffolds into mice is also now being used to mimic the human BM niche, with ceramic scaffolds coated in human mesenchymal stromal cells (MSCs) generating a more human-like BM microenvironment to consequently improve the self-renewal properties of stem cells 51, 69.

The xenograft model of assessing human cell populations in immunocompromised mice is frequently used to study hematologic malignancies, as specific subpopulations of primary CML cells are capable of long-term leukemia-initiating activity 47, 48, 49. The engraftment potential of CD34^+^ cells is commonly tested in sublethally irradiated recipients 12 to 16 weeks following transplant to ensure monitoring of the effects on LT-HSCs only, using D-FISH on remaining cells to distinguish the specificity of drug effects on Ph^+^ and Ph^−^ populations [70] (Fig. 2C). The level of engraftment obtained has been found to correlate with the phase of the disease the patient material is sampled from, with BC cells engrafting at higher rates than those in CP 45, 46. However, such long-term studies can be limiting in terms of time and affordability, and so alternative approaches have also been employed, including examples of engraftment for only 1 week followed by 2 weeks of drug treatment [71] and ex vivo treatment of cells prior to transplant 9, 26. Furthermore, mice engrafted with CML samples do not display the clinical signs of disease associated with other murine CML models, and interpatient variability in CML samples and low levels of engraftment add to the model's limitations. However, despite these technical caveats, the xenograft model of CML remains a gold standard in vivo contribution to preclinical studies. Recent work that illustrated the importance of EZH2 and H3K27me3 reprogramming in LSC survival was able to show the effectiveness of combining an EZH2 inhibitor with nilotinib on Ph^+^CD45^+^CD34^+^CD38^−^ cells from the BM of NSG xenografted mice, being the first published study of CML CP to illustrate efficacy in such a primitive cell population in vivo [10]. The alternative mouse strains now becoming available to researchers in the CML field should make assessment of such primitive populations even more feasible and conducted as standard, and so, going forward, these models are likely to facilitate assessment of the most primitive populations of stem cells responsible for maintaining disease, will reduce animal numbers used, and may also allow development of a clinically characterized leukemia, and thus will provide crucial preclinical information required to generate confidence in therapies progressing toward clinical trials.

## Biomarkers

The transition of potential therapies from first-in-human studies to phase III registration is associated with high rates of attrition. In addition to improvements in preclinical models, the development of appropriate biomarkers, to enable both correct patient stratification and predict drug response, is necessary to improve the progression of drugs through clinical trials and beyond. In CML, the presence of the Ph^+^ chromosome and levels of BCR-ABL transcript can act as chromosomal and molecular markers of disease, respectively, and hematologic, cytogenetic, and molecular responses can thus be monitored in CML patients at defined time points according to principles proposed by European Leukemia Net (ELN) and the National Comprehensive Cancer Network (NCCN) 72, 73. However, prognostic biomarkers that correlate with clinical outcome, and predictive markers of drug resistance, are required to further inform therapeutic strategies. Gene expression profiling through use of microarray has been used in an attempt to identify biomarkers that are predictive of TKI failure, with analysis of CML CD34^+^ cells revealing that genes associated with adhesion are consistently upregulated in TKI nonresponders [74]. Assessment of gene expression changes associated with progression from AP to BC also identified specific gene expression signatures and upregulated signaling pathways, such as the Wnt/β-catenin pathway, with disease progression [75], with such studies providing valuable information to indicate which combinatorial drug choices are most likely to be successful in specific patients. Target-specific biomarkers have been used as commonplace to assess drug response, including assessment of levels of p53, p21, MDM2, Ki-67, and blood macrophage inhibitory factor (MIC-1) for MDM2 inhibitors in liposarcoma [76] and c-Myc mRNA/protein levels and assessment of expression of bromodomain 4-dependent genes following use of BET inhibitors in xenograft models of Burkitt's lymphoma [77]. Although not tested in CML models themselves, the known deregulation of such pathways in CML suggests these biomarkers may also have utility in CML-based models. However, global RNA sequencing following drug treatment in vitro or in vivo can also provide a means of nonbiased screening to interrogate drug action and, thus, can generate a wealth of knowledge for biomarker development. Use of such methods has indicated that drugs designed to stabilize the p53 pathway increase expression of genes associated with apoptosis, whereas inhibitors targeting c-Myc activity can induce transcriptional responses associated with differentiation, in both TKI responder and nonresponder populations [9]. With increased publication of “big data”-based approaches, projects such as Stemformatics are now providing a platform for researchers to access well-curated biological sequence data [78], and so improvements and increased accessibility to nonbiased “omic” approaches will therefore not only improve patient stratification, but will also facilitate prognostic and predictive biomarker identification to inform rationale for treatment selection.

## Systems biology

Integrating computational approaches to model complex biological systems, systems biology is a powerful technique that can be used to enhance our understanding of the complexity of the multiple molecular mechanisms implicated in cancer. Integrating systems-level analysis across multiple cancer types has identified common mechanisms that drive carcinogenesis, including aberrant metabolomics and defective DNA repair 79, 80. Other studies have indicated how systematic analysis of proteomic data can predict drug response and therefore guide therapy selection, including computational approaches in AML, which have predicted sensitivity or resistance to phosphoinositide 3 kinase/AKT inhibition using phosphoproteomic data [81]. The systems biology approach was also recently used to powerful effect in CML, where Abraham, Hopcroft, and colleagues used network analysis of proteomic and transcriptomic data to identify p53 and c-Myc as key hubs that mediate networks to maintain LSCs in CML [9]. Although the identification of p53 and c-Myc as drivers of a cancer phenotype is not a novel concept, with previously described work in the retroviral model being one of many studies to implicate c-Myc in CML progression [63], and studies inhibiting SIRT1 indicating that activation of p53 can enhance elimination of CML LSCs in combination with imatinib mesylate [82], it was the combination of “omic” approaches in normal and CML cells with network analysis that enabled identification of c-Myc and p53 as key regulators of the CML phenotype, despite these targets not being identified as differentially regulated components at the proteomic and transcriptomic level themselves [9]. Combining drug treatments in normal and CML LSCs in vitro with in vivo xenograft NSG and syngeneic SCLtTA/BCR-ABL transgenic models, the authors effectively used numerous preclinical models to illustrate that dual targeting of p53 and c-Myc in CML can synergistically kill and eliminate BCR-ABL^+^ LSCs through increased apoptosis and differentiation. Furthermore, by using drugs already undergoing testing in human clinical trials (Clinicaltrials.gov Identifiers: NCT01773408 and NCT02158858) and ensuring the appropriate networks are still present in TKI nonresponder patients, this package of work combining in vitro, in vivo, and systems-based approaches effectively discovered combinatorial targets that provide promise for future CML therapies.

## Conclusions

To effectively translate exploratory findings into successful therapies, robust and reproducible preclinical models that are predictive of patient response are needed. As in other therapeutic areas, CML research has been advanced through the use of numerous models, ranging from immortalized cell lines to primary patient samples and a variety of murine models. Initially thought of as a classic example of how a genetic abnormality can cause cancer, CML treatment was revolutionized by the advent of TKIs, leading the way in the development of targeted therapies. However, in addition to detection of disease persistence in the clinic, detailed investigations in the described preclinical models have been instrumental in understanding the molecular mechanisms that facilitate the maintenance of CML. Although individual preclinical approaches are often selected based on the hypothesis to be tested, novel combinatorial approaches are able to identify important components of therapeutic interest that might have otherwise been missed using targeted experimental methodologies. Whilst new preclinical models of CML will no doubt be developed in the coming years, to succeed in efficiently progressing therapies through clinical trials and to the patient, it will be the combination and package of preclinical models assembled that will be important in providing the understanding to predict which therapies will be most effective in curing CML.

## Figures and Tables

**Table 1:** Commercially available immortalized CML cell lines^a^

Cell line	Cell type	BCR-ABL status	Derivation
K-562	CML in BC	e14-a2 (b3-a2)	Pleural effusion of a 53-year-old woman with CML in terminal BC (SPI-801 and SPI-802 derived from this line)
KU-812	CML in myeloid BC	e14-a2 (b3-a2)	Peripheral blood of a 38-year-old male patient in BC of CML
Bv-173	B-Cell precursor leukemia	e13-a2 (b2-a2)	Peripheral blood of a 45-year-old man with CML in blast crisis
EM-2/EM-3	CML in BC	e14-a2 (b3-a2)	Sister cell lines established from the BM of a 5-year-old Caucasian girl in second relapse after BM transplant
NALM-1	CML in BC	e13-a2 (b2-a2)	Peripheral blood of a 3-year-old girl with CML
KCL-22	CML in BC	e13-a2 (b2-a2)	Pleural effusion of a 32-year-old woman with CML
LAMA-84	CML in BC	e14-a2 (b3-a2)	Peripheral blood of a 29-year-old woman with CML after onset of myeloid-megakaryocytic BC (LAMA-87 derived from this line)
JK-1	CML in BC	e13-a2 (b2-a2)	Biopsy material of shoulder tumor from 62-year-old man with CML in erythroid blast crisis
MEG-01	CML in megakaryocytic BC	e13-a2 (b2-a2)	BM of a 55-year-old man with CML in megakaryocytic BC
JURL-MK1/MK-2	CML in BC	e14-a2 (b3-a2)	Sister cell lines established from peripheral blood of a 73-year-old man with CML in BC
KYO-1	CML in BC	e13-a2 (b2-a2)	Peripheral blood of a 22-year-old man with CML in myeloid BC
MEG-A2	CML	e14-a2 (b3-a2)	Peripheral blood of a 24-year-old man with CML in megakaryoblastic crisis after chemotherapy
MOLM-1	CML	e13-a2 (b2-a2)	BM of a 41-year-old man with CML in BC
MOLM-6	CML in BC	e13-a2 (b2-a2)	Peripheral blood of a 44-year-old man with CML in BC
TK-6	CML	e14-a2 (b3-a2)	Pleural effusion of a 30-year-old man with CML in T-cell lineage BC after BM transplantation

BC = blast crisis; BM = bone marrow; CML = chronic myeloid leukemia.

a CML cell lines commercially available from DSMZ, including details of their derivation and which variant of the BCR-ABL fusion gene they express, as confirmed by reverse transcription polymerase chain reaction (www.dsmz.de).

**Table 2:** Methods commonly used to assess hematopoietic stem cell cultures

Assay	Principle	Pros	Cons
Colony-forming cell	To study the proliferation and differentiation pattern of hematopoietic progenitors by their ability to form colonies in a semisolid medium	Rapid method for identifying progenitor cells Cells of interest can be harvested from individual colonies for further analysis	Not able to detect more primitive HSCs
Long-term culture initiating cell	Quantification of primitive hematopoietic progenitors Capable of initiating and sustaining myelopoiesis for several weeks in vitro through co-culture methodology	Able to detect primitive HSCs	Time consuming Variation in procedure and stromal cells can influence outcome
Flow cytometry	Passage of cells through a laser to allow the detection of their optical and fluorescence characteristics	Rapid method Able to identify and isolate HSCs and other cells of interest	Surface antigen detection is not functional data
Competitive repopulation	Assessment of the ability of HSCs to serially transplant in immunocompromised mice	In vivo system with more appropriate microenvironment Secondary transplants possible as a true measure of long- term multilineage engraftment	Time consuming Expensive

HSC = hematopoietic stem cell.

**Table 3:** Key discoveries made in CML mouse models and examples of some of the key findings resulting from use of the three commonly used CML mouse models

Model	Key discovery	Reference
Retroviral	CML-like myeloproliferative syndrome can be induced in mice when BCR-ABL-infected BM is transplanted into recipients	38, 39, 40
	Transforming ability of BCR-ABL results from constitutive tyrosine kinase activity	38, 39, 40
	Lineage-restricted target cells and mutational events additional to BCR-ABL expression are required for full malignant transformation	[41]
SCLtTA/BCR-ABL	Expression of BCR-ABL specifically in hematopoietic stem/progenitor cells induces CML-like disease	[42]
	Leukemic phenotype reversed and re-induced by removing and re-introducing tetracycline; thus LSCs are not oncogene addicted	[42]
	BCR-ABL expression induces differentiation and decreases self-renewal capacity of LSK population	[43]
	Disease is transplantable by LSKs, but more severe phenotype is observed after transplantation of unfractionated BM	[44]
Xenograft	Correlation between engraftment in model and disease state of patient material	45, 46
	Identification of specific subpopulations of primary CML cells capable of long-term leukemia-initiating activity	47, 48, 49
	Dynamic role of the BM microenvironment	50, 51

BC = blast crisis; BM = bone marrow; CML = chronic myeloid leukemia; LSC = leukemic stem cell; LSK = Lin^−^Sca-1^+^c-kit^+^.
